# The rationale of using mesenchymal stem cells in patients with COVID‐19‐related acute respiratory distress syndrome: What to expect

**DOI:** 10.1002/sctm.20-0164

**Published:** 2020-07-21

**Authors:** Alp Can, Hakan Coskun

**Affiliations:** ^1^ Laboratory for Stem Cells and Reproductive Cell Biology, Department of Histology and Embryology Ankara University Faculty of Medicine Ankara Turkey; ^2^ Harvard Medical School Boston Massachusetts USA; ^3^ Department of Cardiology Boston Children's Hospital Boston Massachusetts USA

**Keywords:** acute respiratory distress syndrome, clinical trial, COVID‐19, immunomodulation, inflammatory disease, mesenchymal stem cells

## Abstract

The severe acute respiratory syndrome coronavirus 2 (SARS‐CoV‐2)‐caused coronavirus disease 2019 (COVID‐19) pandemic has become a global health crisis with an extremely rapid progress resulting in thousands of patients who may develop acute respiratory distress syndrome (ARDS) requiring intensive care unit (ICU) treatment. So far, no specific antiviral therapeutic agent has been demonstrated to be effective for COVID‐19; therefore, the clinical management is largely supportive and depends on the patients' immune response leading to a cytokine storm followed by lung edema, dysfunction of air exchange, and ARDS, which could lead to multiorgan failure and death. Given that human mesenchymal stem cells (MSCs) from various tissue sources have revealed successful clinical outcomes in many immunocompromised disorders by inhibiting the overactivation of the immune system and promoting endogenous repair by improving the microenvironment, there is a growing demand for MSC infusions in patients with COVID‐19‐related ARDS in the ICU. In this review, we have documented the rationale and possible outcomes of compassionate use of MSCs, particularly in patients with SARS‐CoV‐2 infections, toward proving or disproving the efficacy of this approach in the near future. Many centers have registered and approved, and some already started, single‐case or phase I/II trials primarily aiming to rescue their critical patients when no other therapeutic approach responds. On the other hand, it is also very important to mention that there is a good deal of concern about clinics offering unproven stem cell treatments for COVID‐19. The reviewers and oversight bodies will be looking for a balanced but critical appraisal of current trials.

## Graphical abstract

The therapeutic potential of mesenchymal stem cells in acute respiratory distress syndrome‐developed COVID‐19 cases was tested in recently approved clinical trials. They possess several features facilitating the resorption of the alveolar exudate by type I and type II alveolar cells and improving the pulmonary microenvironment, thus recovering the lung functions in COVID‐19 patients.

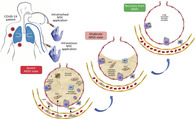

**Significance statement**

Although mesenchymal stem/stromal cell (MSC) administration is an unproven stem cell therapy approach for COVID‐19 patients, there is a growing demand for new therapies among patients and healthcare workers. Due to preclinical findings and few current clinical data set, MSCs possess remarkable immunomodulatory features, and thus have the potential to recover the pulmonary microenvironment, intercept pulmonary fibrosis, and cure lung dysfunction in COVID‐19 pneumonia and respiratory distress syndrome. However, it is also noteworthy to point out that there is a good deal of concern about clinics offering unproven stem cell treatments for COVID‐19, and reviewers will be looking for a balanced but critical appraisal of current trials.

## 1 INTRODUCTION

The coronavirus disease 2019 (COVID‐19) pandemic, caused by a novel beta coronavirus named severe acute respiratory syndrome coronavirus 2 (SARS‐CoV‐2), possessing a single‐strand, positive‐sense RNA genome (26‐32 kilobases in length),^1^ is the definition of the greatest health challenge of our times. Since its emergence in Guangdong, Southern China, late last year, the virus has spread to 213 countries in every continent except Antarctica as of late June 2020.^2^, ^3^ Cases are still rising, especially in Russia, Brazil, the United States, and the United Kingdom, even though a declining trend was noted in deaths in recent weeks. Countries are struggling with slowing down the spread of the virus by testing and treating patients, quarantining infected citizens, limiting or banning leisure travel, prohibiting large social gatherings, and closing schools. As of 23 June 2020, more than 9.2 million cases have been reported worldwide with approximately 475 000 deaths.^3^ The case fatality rate (CFR) varies from 2.3% to 14.8% depending on the demographics of the nation or region, age, severity of the disease, and comorbidities. Overall, the CFR was reported to be approximately 11% worldwide among COVID‐19‐positive cases, and the ratio of serious or critical cases is approximately 2%. Older adults between the ages of 70 and 80 years have a CFR of 8.0%, and those aged more than 80 years have a CFR of 14.8%.^4^ The CFR was reported to 49.0% among critical cases and was significantly high among those with preexisting comorbid conditions—10.5% for cardiovascular disease, 7.3% for diabetes, 6.3% for chronic respiratory disease, 6.0% for hypertension, and 5.6% for cancer.^4^ In contrast, more than 50% of children (younger than 18 years) experienced mild symptoms or were asymptomatic, with fewer than 6% of children developing severe symptoms.^5^

COVID‐19 has an incubation period of 2 to 14 days; the mean incubation period is 5.2 days.^6^, ^7^ Although approximately 30% of infected people are asymptomatic, the onset of illness is characterized by a series of clinical symptoms from mild to severe, including fever (98% of patients), cough, shortness of breath and/or chest pain (76%), and myalgia or fatigue (44%).^8^ Less common symptoms are sputum production, sore throat, loss of taste and smell, headache, hemoptysis, and diarrhea. Therefore, disease stage is classified according to the patient's clinical symptoms and laboratory findings as (a) *mild type*: mild clinical symptoms without pneumonia; (b) *common type*: fever, respiratory tract and other symptoms with pneumonia; (c) *severe type*: respiratory distress (respiratory rate is higher than 30 times per minute; in resting state, oxygen saturation is lower than 93%; partial pressure of oxygen [PaO_2_] to fraction of inspired oxygen [FiO_2_] ratio lower than 300 mmHg); (d) *critical type*: respiratory failure requiring mechanical ventilation, shock, and other organ failure requiring intensive care unit (ICU) monitoring and treatment.^9^ In addition, patients with acute cardiac injury present with tachycardia or bradycardia. Critically ill individuals may also have acidosis and increased lactate.^6^

No specific antiviral therapeutic agents or vaccines for COVID‐19 have been proven to be effective so far. Several therapies, such as ribavirin, remdesivir, favipiravir, and oseltamivir, are under investigation,^4^, ^10^ but the antiviral efficacy of these drugs is not yet known. The supportive approach includes corticosteroids, antibiotics, anticoagulants, and oxygen therapy. Recently, convalescent plasma therapy for COVID‐19 has been under consideration to prove its safety and efficacy. Promising results (32 participants) and 48 ongoing trials were accumulated in a Cochrane analysis.^11^

As defined below, cytokine storm (high serum levels of granulocyte‐colony stimulating factor [GSCF], interferon‐γ inducible protein 10 [IP‐10], monocyte chemoattractant protein 1 [MCP‐1], macrophage inflammatory protein [MIP]‐1A, interleukin [IL]‐2, IL‐6, IL‐7, and tumor necrosis factor [TNF]) in COVID‐19‐infected patients results in pulmonary edema, dysfunction of air exchange, acute respiratory distress syndrome (ARDS), and acute cardiac injury and may lead to death. As mesenchymal stem cells (MSCs) possess many remarkable immunomodulatory and extracellular matrix remodeling effects through secretion of several types of cytokines, growth factors, and tissue mediators, they modulate the cytokine storm or balance immune responses as one of their therapeutic effects in recovering the pulmonary microenvironment, protecting alveolar epithelial cells, intercepting pulmonary fibrosis, and curing lung dysfunction and COVID‐19 pneumonia. In this review, we endeavored to discuss the potentials of MSC‐based cell therapies in the light of current literature obtained from both preclinical and clinical studies.

## 2 DIAGNOSIS AND INFLAMMATORY PROFILE OF PATIENTS WITH COVID‐19

The diagnosis of COVID‐19 is performed by the detection of the virus in respiratory secretions (throat, nasopharynx, sputum, and endotracheal aspirates and bronchoalveolar lavage) by sensitive antigen tests such as polymerase chain reaction assay, even though the testing efficiency is around 60% to 70% in sputum and nasal swab samples.^12^ Besides common radiographic tests such as x‐ray and computed tomography (CT) imaging, more sophisticated serologic and genomic tests are also available including the full genome analysis by next‐generation sequencing.^13^ As summarized in Table 1, blood test findings include normal/low leukocyte counts with high C‐reactive protein (CRP). There may be lymphopenia; fewer than 1000 lymphocytes has been associated with severe disease. The platelet count is usually normal or slightly low. The CRP and erythrocyte sedimentation rate are generally elevated; however, procalcitonin levels are usually normal. A high procalcitonin level may imply a bacterial coinfection. The aspartate transaminase (AST) and alanine transaminase (ALT), prothrombin time, creatinine, d‐dimer, creatinine phosphokinase, and lactate dehydrogenase may be elevated, and high levels are associated with severe disease.^14^, ^15^ CT chest scans are usually abnormal even in those with no symptoms or mild disease. However, starting from the common cases, CT imaging shows infiltrates, ground‐glass opacities, and subsegmental consolidation. It may also have abnormal results in asymptomatic patients or patients with no clinical evidence of lower respiratory tract involvement. In fact, abnormal CT scans have been used to diagnose COVID‐19 in suspected virus‐negative cases; many of these patients become positive on repeat testing.^16^

**TABLE 1:** The immunological, serological, and histopathological profile of patients with COVID‐19

Immunopathology	Serum/blood tests	Lung histopathology
IFN‐γ ↑, IL‐1β ↑, IP‐10 ↑, MCP‐1 ↑, GCSF ↑, IL‐2 ↑, IL‐6 ↑, IL‐7 ↑, IL‐8 ↑, IL‐9 ↑, IL‐10 ↑, IL‐17 ↑, MIP‐1α ↑, MCP‐1 ↑, IP‐10 ↑, TNF‐α ↑	AST ↑, ALT ↑, LDH ↑, CPK ↑, creatinine ↑, CRP ↑, ESR ↑, γ‐GT ↑, α‐HBDH ↑, d‐dimer ↑, total bilirubin ↑, ferritin ↑, prothrombin time ↑, cardiac troponin ↑, procalcitonin ↑, albumin ↓, hemoglobin ↓, eosinophils ↓, basophils ↓, neutrophils ↑, monocytes ↓, CD14^+^ CD16^+^ monocytes ↑, lymphocytes ↓	Atypical enlarged pneumocytes and/or desquamation of pneumocytes Hyaline membrane formation Cellular or proteinaceous exudates Alveolar hemorrhage Fibrinoid necrosis of small vessels Multinucleated syncytial cells Interstitial accumulation of mononuclear cells (monocytes and T cells) Endothelial dysfunction associated with apoptosis

*Note*: The degree of elevation (↑) or decline (↓) and histopathological changes vary by the severity of patient's clinical status from mild to severe cases of acute respiratory distress syndrome.

Abbreviations: α‐HBDH, α‐hydroxybutyrate dehydrogenase; γ‐GT, gamma‐glutamyl transpeptidase; ALT, alanine transaminase; AST, aspartate transaminase; CPK, creatine phosphokinase; CRP, C‐reactive protein; ESR, erythrocyte sedimentation rate; GCSF, granulocyte‐colony stimulating factor; IFN‐γ, interferon gamma; IL, interleukin; IP‐10, IFN‐γ inducible protein 10 (also known as CXC‐chemokine ligand 10); MCP‐1, monocyte chemoattractant protein 1 (also known as CC‐chemokine ligand 2); LDH, lactic acid dehydrogenase; MIP‐1α, macrophage inflammatory protein 1α (also known as CC‐chemokine ligand 3); TNF‐α, tumor necrosis factor‐α.

According to the laboratory tests published so far, in a subset of patients with COVID‐19, clinical progress is associated with an activation of an inflammatory cascade, called “cytokine storm syndrome,” which is mainly due to activated T‐helper 1 (Th1) and T‐helper 17 (Th17) cell responses.^17^, ^18^ It is a diverse set of conditions unified by a clinical phenotype of systemic inflammation, multiorgan failure, and hyperferritinemia^19^ and is associated with a wide variety of virus infections, such as severe acute respiratory coronaviruses (including SARS‐CoV‐2), influenza virus, and dengue virus, and noninfectious diseases.^20^ Some patients with COVID‐19 progress to this hyperinflammatory condition, often with pulmonary involvement resembling the secondary hemophagocytic syndrome, which is commonly triggered by viral infections.^21^ This systemic hyperinflammation results in inflammatory lymphocytic and monocytic infiltration of the lung and heart, causing ARDS and cardiac failure. Huang et al^8^ noted that patients with COVID‐19 both in the ICU and not requiring the ICU demonstrated higher cytokine profiles compared with healthy adults, characterized by increased IL‐1β, IL‐2, IL‐7, IL‐8, IL‐9, IL‐10, IL‐17, GCSF, IP‐10, MCP‐1, MIP‐1α, TNF‐α, and some growth factors such as platelet‐derived growth factor and vascular endothelial growth factor (VEGF)^8^ (Table 1). Huang et al further reported that plasma concentrations of IL‐2, IL‐7, IL‐10, GCSF, IP‐10, MCP‐1, MIP‐1α, and TNF‐α were higher in patients in the ICU than in patients not in the ICU. On the other hand, plasma levels of IL‐5, IL‐12p70, IL‐15, eotaxin, and RANTES were found to be comparable between healthy adults and infected patients. Predictors of mortality in a recent retrospective study of 150 confirmed COVID‐19 cases in Wuhan, China, included elevated IL‐6.^22^ Taken together, cytokine profiles of patients seem to lead to mortality that is directly related to virus‐driven hyperinflammation.^23^ As demonstrated in patients with severe acute respiratory syndrome (SARS) more than 15 years ago, elevated levels of T‐helper cell‐originated proinflammatory cytokines, that is, interferon (IFN)‐γ, IL‐1B, IL‐6, IL‐12, IP‐10, neutrophil‐originated chemokine IL‐8, and MCP‐1, were associated with pulmonary inflammation and extensive lung damage.^24^ More recently, Mahallawi et al reported marked increases in the concentrations of IFN‐γ, TNF‐α, IL‐15, and IL‐17 in patients with Middle East respiratory syndrome (MERS) coronavirus compared with controls.^25^ Secretion of such cytokines and chemokines attracts immune cells, notably monocytes and T lymphocytes (CD4+ T cells), but not neutrophils, from the blood into the infected site. Pulmonary recruitment of immune cells from the blood and the infiltration of lymphocytes into the airways may explain the lymphopenia and increased neutrophil‐lymphocyte ratio seen in around 80% of patients with SARS‐CoV‐2 infection.

Besides the cytokine storm, COVID‐19‐related clinical manifestations reflect that patients develop lymphocytic endotheliitis as evidenced by the impairment of many organs, including kidney, heart, small intestine, and lungs.^26^ Therefore, COVID‐19 pathogenesis in the endothelial cells causes a vascular disease state during which 89% of hospitalized patients showed subsegmental vascular enlargement on their initial CT scans.^27^ Studies have also reported evidence of a COVID‐19‐associated coagulopathy. It has been shown that 90% of patients with pneumonia had increased coagulation activity, marked by elevated d‐dimer, a fibrin degradation thromboembolic marker^28^ predicting a poor prognosis in COVID‐19.

## 3 ARDS IN COVID‐19

ARDS, by definition, is a clinical state characterized by an acute presentation of severe and refractory hypoxemia, decreased airway compliance, microscopic evidence of diffuse alveolar damage, and bilateral pulmonary infiltrates excluding cardiac‐related edema.^29^ Respiratory failure from ARDS has been reported as the leading cause of mortality in COVID‐19 cases.^22^ Around 10% to 20% of patients with severe COVID‐19 may develop shortness of breath, frequently in the second week of the disease, which might be accompanied by or progress to hypoxemia.^8^ The mean period from onset of symptoms to dyspnea was reported as 5 days, hospitalization 7 days, and the onset of ARDS 8 to 9 days.^14^ Respiratory damage will inevitably progress into ARDS, defined as PaO_2_/FiO_2_ lower than 300 mmHg, during days 8 to 14 of the illness, as well as resulting in noncardiogenic pulmonary edema and mechanical ventilation.^8^, ^30^

ARDS constitutes an acute hypoxemic respiratory failure that originates mainly from an increase in lung endothelial and epithelial permeability, which result in outflow of fluid into alveoli, leading to noncardiogenic pulmonary edema and decreased arterial oxygenation. Damage to the lung parenchyma results from multiple mechanisms, including direct injury by the inflaming agent (eg, bacteria and their products, viral invasion, and acid injury after aspiration), by harm in resulting from hyperactivation of the immune system, and by mechanical stretch‐induced damage caused by mechanical ventilation.^31^ The pathologic features of ARDS in alveoli and their microenvironment in COVID‐19 greatly resemble those seen in SARS and MERS infections,^32^, ^33^ including uni/bilateral diffuse alveolar parenchyma damage with cellular fibromyxoid exudates, desquamation of pneumocytes, and hyaline membrane formation^34^ (Table 1). Mononuclear infiltrates—predominantly by lymphocytes—are frequently seen in interalveolar septa. Multinucleated syncytial cells with enlarged alveolar cells characterized by large nuclei, granular cytoplasm, and prominent nucleoli are identified in the intra‐alveolar septa,^34^ directly presenting the viral cytopathic changes. There may be no obvious intranuclear or intracytoplasmic viral inclusions.

## 4 MESENCHYMAL STEM CELLS

MSCs are fibroblast‐like precursors resident in most adult and neonatal tissues. MSCs are isolated based on their ability to adhere to plastic culture dishes, and they are capable of proliferating by in vitro passaging. They are characterized by their capacity to differentiate into cells of mesodermal origin, such as adipocytes, chondroblasts, and osteoblasts,^35^ and they possess certain cell surface molecules: CD73, CD90, and CD105.^36^ Although much debate remains as to how to define such a wide population of cells, it is apparent that some populations of MSCs are capable of demonstrating stem cell functions both in vivo and in vitro.^37^ In addition to their stem or progenitor properties, MSCs have also been shown to possess extensive immunoregulatory abilities and are capable of manipulating both adaptive and innate immune responses.^38^ Clues obtained from both preclinical and clinical studies have demonstrated that MSCs actively interact with components of the innate immune system and that, through these interactions, they display both anti‐inflammatory and proinflammatory effects.^39^ Paracrine effects of MSCs were reported several years ago by Haynesworth et al,^40^ who demonstrated that MSCs synthesize and secrete a wide range of growth factors and cytokines such as VEGF, fibroblast growth factor (FGF), MCP‐1, hepatocyte growth factor (HGF), IGF‐I, stromal cell‐derived factor 1, and thrombopoietin, which exert actions on their microenvironment. Therefore, MSCs constitute an important resource for cellular therapy. They have been the subject of clinical trials as a cellular pharmaceutic agent since 1995^41^ and have become the central point of the most clinically studied experimental cell therapy in the world.^42^, ^43^ However, only a small number of studies investigating the effects of MSCs in preclinical models of respiratory virus infections were published so far (reviewed by Khoury et al^44^), and no established animal model for coronavirus respiratory infection exists to investigate the effects of MSC administration in nonhuman controlled experiments. Severe acute respiratory coronavirus replication was observed in *STAT1* ^−/−^ knockout mice after intranasal infection, but this model failed to show clinical signs of pulmonary disease as seen in human.^45^

## 5 MSC SOURCES

MSCs can be isolated from various tissue sources. The selection of the source is based on their logistical, practical, and in vitro characteristics. Currently, the main sources of MSCs are bone marrow, umbilical cord stroma, adipose tissue, and dental pulp.

### 5.1 Bone marrow

Bone marrow mesenchymal stem cells (BM‐MSCs) are multipotent cells that are able to differentiate into mesodermal lineage.^46^ They are isolated from bone marrow aspirates and subsequently expand in vitro. They have been shown to express a variety of cell surface markers, including CD44, CD73, CD105, and CD146, but they lack hemopoietic markers CD11b, CD14, CD34, and CD45.^47^ BM‐MSCs have inherently low immunogenicity because of the low expression of major complex of histocompatibility (MHC) class I and the lack of MHC class II expression. They also do not express the costimulatory molecules CD40 and CD80. They inhibit T‐lymphocyte proliferation and activation in response to alloantigens.^48^ Collectively, the low immunogenicity properties of BM‐MSCs and the interactions between BM‐MSCs and immune cells provide BM‐MSC‐mediated induction of tolerance that could be therapeutic for modulation of inflammation.

### 5.2 Umbilical cord

Human umbilical cord stroma, which is known as Wharton's jelly, is a rich source of primitive mesenchymal stromal cells (UC‐MSCs or WJ‐MSCs). UC‐MSCs are multipotent cells that can be differentiated into mesodermal as well as to neuronal lineages.^49^ UC‐MSCs are harvested from the umbilical cord stroma using mechanical or enzymatic techniques^50^ and subsequently expanded in vitro. However, an explant culture system provides a more physiologic milieu to harvest intact cells compared with enzymatic isolation.^51^ UC‐MSCs are positive for CD73, CD90, CD105, and human leukocyte antigen (HLA) class I and negative for CD45 and HLA‐DR.^36^, ^52^ They display immunosuppressive potential by cell‐cell contact with activated T cells,^53^ and they efficiently suppress the proliferation and cytokine production of a subset of circulating T cells.^54^ Moreover, UC‐MSCs produce large amounts of tolerogenic IL‐6, transforming growth factor (TGF)‐β, and VEGF‐1.^55^ Given that UC‐MSCs exhibit many unique features, involving the regulation of both innate and adaptive immune responses,^56^ they stand as the most widely used and first‐selected allogeneic MSCs in several immunocompromised diseases.^57^, ^58^

### 5.3 Adipose tissue

Several studies revealed that adipose tissues contain MSCs, termed adipose tissue‐derived stem cells (ADSCs). They are preferentially located in the stromal vascular fraction of isolated adipose explants. In conditional cultures, ADSCs exhibit a multipotent differentiation capacity into cell types originated from three germ layers.^59^ ADSCs express CD44, CD73, CD90, and CD105 and are negative for CD31 and CD45.^60^ ADSCs can induce proliferation of IL‐10‐producing regulatory B cells that regulate the immune system by anti‐inflammatory potential.^61^ Therefore, ADSCs can be used in the treatment of diverse immune‐related disorders, including graft‐vs‐host disease.^62^ As the isolation of autologous ADSCs is relatively simple and less invasive compared with BM‐MSCs, they have been the first choice in many trials. However, ADSCs still exhibited only moderate benefits in clinical trials.

### 5.4 Dental pulp

Dental pulp MSCs (DP‐MSCs), primarily isolated from the dental pulp,^63^ can also be isolated from deciduous teeth, apical papilla, periodontal ligament, dental follicles, and gingiva.^64^ DP‐MSCs express CD29, CD73, CD90, and CD105 and do not express the hematopoietic stem cell markers CD11b, CD14, CD34, CD45, and HLA‐DR. They have the capacity to differentiate into mesodermal lineage under certain conditions in vitro.^36^ However, DP‐MSCs also express stemness‐related markers similar to embryonic stem cells, such as Oct‐3/4, Nanog, and Sox‐2.^65^ Like MSCs from other tissues, DP‐MSCs also have a strong immunomodulatory ability with a higher suppression rate of T‐lymphocyte growth capacities.^66^ Moreover, DP‐MSCs can suppress lymphocyte proliferation and increase the number of regulatory T cells and IL‐10 while decreasing IL‐4 and IFN‐γ levels.^67^ Therefore, dental pulp is considered a novel MSC source to use for the treatment of autoimmune and inflammatory diseases.

## 6 IMMUNOREGULATORY FEATURES OF MSCS

Studies suggest that the tissue from which MSCs originate determines their capacity to differentiate and their immunoregulatory potential.^68^ However, variations in the range and functional status of immune cells in the inflammation site also affect the ability of MSCs to regulate inflammation. MSCs express receptors for chemokines, through which they sense and migrate toward inflammatory signals, such as complement factors C3a and C5a, which are released by damaged tissues^69^ and serve as chemoattractants for MSCs. An active state of inflammation is often subject to dynamic changes, which can modify the immunologic properties of MSCs. Thus, anatomic location and inflammatory state are critical determinants of the immunoregulatory properties of MSCs and might govern the therapeutic potential of these cells in inflammatory diseases.

Several kinds of innate immune cells, like neutrophils, mast cells, macrophages, myeloid‐derived suppressor cells, dendritic cells, and natural killer (NK) cells, exist at inflammation sites and can be synchronized by MSCs, whereas variations in chemokine expression can dictate the MSC functions. For instance, macrophages exist in a spectrum of different phenotypes as proinflammatory (M1) or anti‐inflammatory (M2), both of which expresses their functional states and are associated with distinct pathologies. MSCs facilitate monocyte‐to‐macrophage transition, potentiate their microbicidal responses, skew naïve macrophages to a M1 state, attenuate already activated M1 macrophages, and enhance M2 activation. This polarization of macrophages from M1 to M2 state is mediated by the production of MSC‐derived immunosuppressive molecules and metabolites, such as prostaglandin E2 (PGE_2_)^70^ and TNF‐stimulated gene 6 protein (TSG6).^71^ Elucidation of key monocyte and macrophage metabolic programs in monocyte‐MSC, M1‐MSC, and M2‐MSC cocultures indicated changes in glucose transporter‐1 expression/glucose uptake, indoleamine 2,3‐dioxygenase (IDO) 1 protein/activity, SIRTUIN1, and alterations in activated‐protein kinase and mammalian target of rapamycin activity, reflecting MSC‐instructed metabolic shifts.^70^ MSCs could also inhibit the infiltration of macrophages, monocytes, and neutrophils into sites of inflammation in a TSG6‐dependent mode. This mechanism is critical to the ability of MSCs to alleviate acute lung injury.^72^ Inflammatory cytokines that control chemokine production can provoke MSCs to recruit monocytes, macrophages, and neutrophils to their proximity. Similarly, inflammation can induce the expression of IDO by MSCs, with immunosuppressive consequences on myeloid cell migration. These findings elucidate the complexity of MSC‐innate immune cell interactions in an inflammatory microenvironment and the difficulty in predicting whether the immunomodulatory response of MSCs tend to be positive or negative.

**FIGURE 1:**
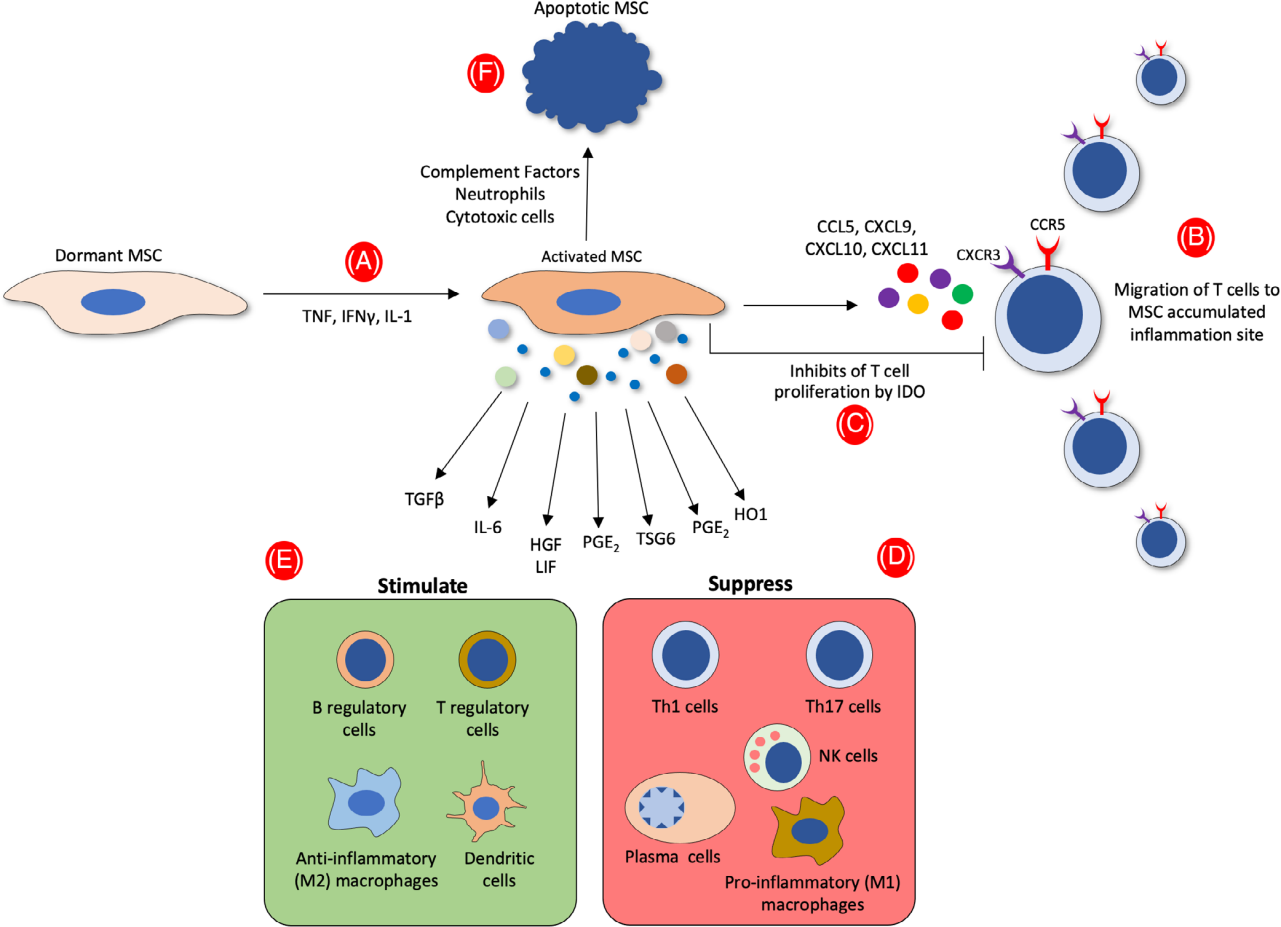
Interactions of MSCs with their microenvironment after inflammation is triggered. A, MSCs produce TNF, IFN‐γ, and IL‐1 in the proinflammatory state and consequently acquire an activated state that stimulates two main consecutive events. B, Recruitment of T cells to the vicinity of MSCs by the secretion of CCL5, CXCL9, CXCL10, CXCL11, which then bind to their de novo synthesis of specific receptors (CXCR3 and CCR5). C, Suppression of the proliferation and activity of T cells in their vicinity by IDO secretion. Immunosuppression by MSCs is also mediated by the production of TGF‐β, IL‐6, HGF, leukemia inhibitory factor (LIF), PGE_2_, TSG6, and HO1 exhibited by several sizes of extracellular vesicles. D, E, These factors stimulate the proliferation of anti‐inflammatory immune cells (anti‐inflammatory M2 macrophages, regulatory T cells, and B cells) while inhibiting the proliferation and function of proinflammatory immune cells (Th1 and/or Th17 cells, proinflammatory M1 macrophages, neutrophils, NK cells, and B cells). F, Transplanted MSCs may be assaulted by complement factors, complement‐activated neutrophils, and cytotoxic cells, all of which will induce them to undergo apoptosis. CCL5, CC‐chemokine ligand 5; CCR5, CC‐chemokine receptor 5; CXCL, CXC‐chemokine ligand; CXCR3, CXC‐chemokine receptor 3; HGF, hepatocyte growth factor; HO1, heme oxygenase 1; IDO, indoleamine 2,3‐dioxygenase; IFN, interferon; IL, interleukin; LIF, leukemia inhibitory factor; MSC, mesenchymal stem cell; NK, natural killer; PGE_2_, prostaglandin E2; TGF, transforming growth factor; Th1, T‐helper 1; Th17, T‐helper 17; TNF, tumor necrosis factor; TSG6, TNF‐stimulated gene 6 protein

The immunosuppression cascade starts with the induction of MSCs by IFN‐γ, which, combined with either TNF or IL‐1, costimulates the production of CC‐chemokine receptor 5 and CXC‐chemokine receptor 3 ligands, including CC‐chemokine ligand 5, CXC‐chemokine ligand (CXCL) 9, CXCL10, and CXCL11, all of which attract T cells to the proximity of MSCs (extensively reviewed by Shi et al^68^). Subsequently, MSCs in humans suppress the proliferation and activity of T cells in their vicinity by expressing IDO (Figure 1). Immunoregulation by MSCs is also mediated by the production of cytokines (TGF‐β and IL‐6), growth factors (HGF and leukemia inhibitory factor), and anti‐inflammatory mediators (PGE_2_, TSG6, heme oxygenase 1, galectins, and extracellular vesicles [EVs]). These factors inhibit the proliferation and function of proinflammatory immune Th1 and/or Th17 cells, proinflammatory M1 macrophages, neutrophils, NK cells, and B cells and enhance the numbers of anti‐inflammatory immune cells, including anti‐inflammatory M2 macrophages, regulatory T cells, and regulatory B cells. Anti‐inflammatory immune cells can further suppress the activity and functions of proinflammatory cells and thus promote tissue reparation and regeneration. On the other hand, transplanted MSCs can be attacked by factors of the complement system, complement‐activated neutrophils, and perforin‐positive cytotoxic cells, stimulating them to undergo apoptosis. Apoptotic MSCs can then be engulfed by macrophages so they induce the phagocytes to express IDO, with immunosuppressive consequences.

Interestingly, tissue‐resident MSCs may not be as effective as transplanted MSCs in restoring immunologic homeostasis in damaged tissues.^68^ One possible explanation for this difference is that in vitro cultured and expanded MSCs are often administered in large quantities (typically 1 × 10^6^ cells per kilogram of body weight) compared with a normal concentration of less than 0.05% stromal cells among the entire population of bone marrow cells.^73^ Another possible scenario is that MSCs may be changing their immune properties during in vitro expansion.

## 7 ANTIFIBROTIC (EXTRACELLULAR MATRIX REMODELING) PROPERTIES OF MSCs

Healthy tissues can be damaged after acute or chronic stimuli, such as mechanical or chemical injuries, infections, or autoimmune reactions. Dead or damaged cells and extracellular matrix (ECM) elements are replaced by the formation of scar tissue composed of stiff cross‐linking collagen fibers mostly synthesized by myofibroblasts; thus, this process ensures organ functionality, although usually of inferior quality. In the last stage or as an alternative path of the repair process, fibrosis, also known as fibrotic scarring, replaces scarred parenchymal tissue to a great extent, leading to considerable tissue remodeling and the formation of permanent scar tissue. However, unrestricted fibrosis leads to substantial remodeling of the ECM with pathologic features. In most cases, this process leads to perturbed organ function and death. It has been demonstrated that matrix metalloproteinases (MMPs, a group of proteolytic enzymes that are capable of degrading all types of extracellular matrix proteins: MMP3, MMP6, and MMP9) and tissue inhibitor of matrix metalloproteinases (TIMPs) TIMP1 and TIMP3 decreased after MSC treatment of pressure overload hypertrophy.^74^ Moreover, administration of MSCs prevented irradiation‐induced lung fibrosis by reducing production of inflammatory cytokines, proliferation of fibroblasts, and accumulation of collagen.^75^ They also limit the fibrotic response by reducing myofibroblast differentiation from epithelial cells and fibroblasts.^76^ Thus, MSCs are considered as critical elements in ECM remodeling after tissue damage; therefore, they represent a therapeutic tool in recovering normal tissue functions in fibrosis.

## 8 MSC‐DERIVED EVS

Recent studies suggest that the signals responsible for the therapeutic effects of MSCs are at least partially linked to the production of EVs. Simply, EVs are small, round packages Type‐I alveolar cell of secretory products enclosed by a phospholipid bilayer membrane, which carry several bioactive components.^77^ Although confusion on the nomenclature of EVs exists in the literature, EVs are currently classified into exosomes, microvesicles, and apoptotic bodies according to their cellular origin.^78^ EVs encapsulate cargo molecules, including proteins, microRNA, and mRNAs, from their cell of origin.^79^ Furthermore, they carry several types of membrane proteins, including glycosylated adhesion molecules and receptors, and lipids.^80^, ^81^ A huge number of studies demonstrated that MSCs could modulate immune response by cell‐to‐cell contact, secretion of soluble factors, induction of anergy, apoptosis, and regulatory immune cells to induce anti‐inflammatory signals and immune tolerance states.^82^, ^83^ Among those, MSC‐derived EVs play a role in the regulation of the inflammatory response by increasing the level of anti‐inflammatory cytokines and decrease proinflammatory cytokines, which opens avenues to promising immune therapy approaches. A recent study demonstrated that MSC‐derived EV cargoes comprised immunomodulatory cytokines such as IFN‐γ‐induced protein 10, MIP‐1β, IL‐8, chemokine growth‐regulated oncogene, and TIMP1.^84^ Moreover, cytokines, including intercellular adhesion molecule‐1, basic FGF, CD105, and a microRNA (miR210), which has roles in vascular repair signaling, were also identified from MSC‐derived EVs. However, there are several challenges and potential risk factors that should still be considered, including the difficulties of harvesting and purifying EVs from various types of samples (culture media, serum, urine, milk, and other potential sources).^85^ The safety, immunogenic response potency, and optimal doses of EVs are still largely unknown. There are a limited number of clinical studies employing EV‐based therapies focused on neurologic, cardiovascular, liver, kidney, and skin diseases (ClinicalTrials.gov). Nonetheless, MSC‐EV‐based therapies present fundamental advances in terms of potential cell‐free therapy options in the future.

## 9 MSC‐DRIVEN MITOCHONDRIAL TRANSFER

**FIGURE 2:**
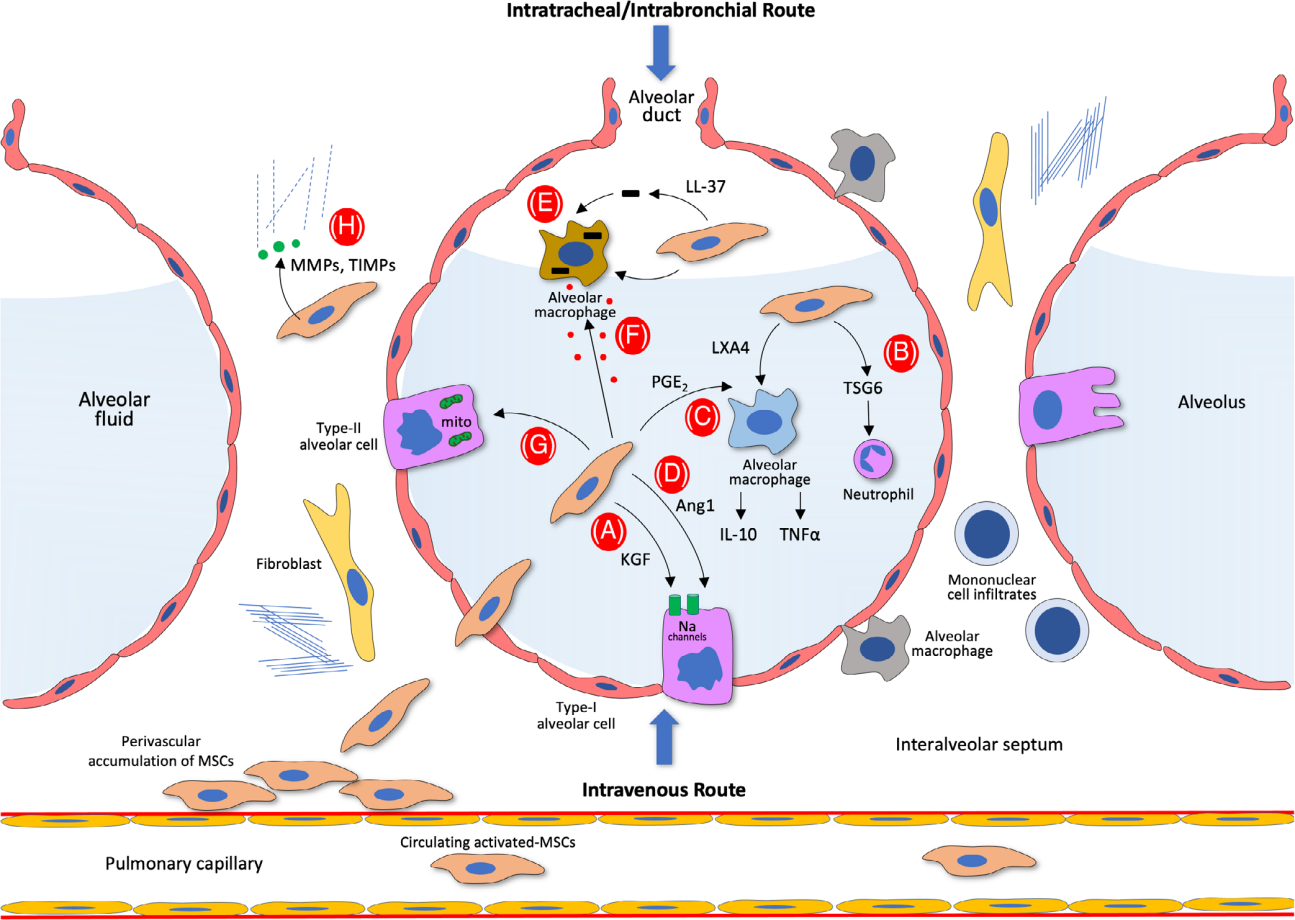
Alveolar microenvironment in acute respiratory distress syndrome (ARDS) and MSC‐mediated repairing pathways. Alveolar injury is characterized by the impairment of endothelial and type I alveolar cell (pneumocyte) barrier, which eventually results in an intense accumulation of proteinaceous fluid (alveolar edema) and mononuclear cell (lymphocytes) infiltrates in interalveolar septa. Extravasated and/or resident MSCs can trigger a series of direct and indirect repairing mechanisms. A, Clearance of the increased alveolar edema can be induced by the release of KGF by enhancing sodium‐dependent alveolar fluid clearance through type II alveolar sodium channels. B, MSC‐released TSG6 decreases neutrophil functions, which directly affects improvement of the vascular endothelial and alveolar epithelial barriers. C, Resolution of inflammation can be further enhanced by the increased release of IL‐10 and decreased release of TNF‐α, which are mediated by LXA4 and PGE_2_. D, Increased epithelial repair in type II alveolar cells can be restored by the release of Ang1. (E, F, MSCs can also facilitate the phagocytosis of bacteria by the intra‐alveolar and interalveolar macrophages by releasing the antimicrobial peptide LL‐37 or by the transfer of extracellular vesicles to macrophages from MSCs. G, Additionally, MSCs can exert their actions through mitochondrial transfer to injured alveolar cells, thus increasing their ATP content, which would improve bioenergetics and increase alveolar epithelial function, improving surfactant release by type II alveolar cells. H, MSCs can degrade or inhibit ARDS‐induced fibrotic tissue formation (collagen fiber accumulation) to modulate the extracellular matrix by releasing MMPs and TIMPs. In MSC‐based therapies, infusion of auto/allogeneic MSCs are applied through two primary routes (ie, intravenous and intratracheal/intrabronchial). Ang1, angiopoietin‐1; IL, interleukin; KGF, keratinocyte growth factor; LXA4, lipoxin A4; MMP, matrix metalloproteinase; MSC, mesenchymal stem cell; PGE_2_, prostaglandin E2; TIMP, tissue inhibitor of matrix metalloproteinase; TNF, tumor necrosis factor; TSG6, TNF‐stimulated gene 6 protein. *Source*: Laffey and Matthay, 2017^92^

MSCs have been reported to present protective effects in several preclinical experimental models of ARDS, including the ex vivo human lung perfusion model.^86^, ^87^, ^88^ However, the mechanism of this effect was largely unknown. Initially, Islam et al showed that MSCs could transfer mitochondria to impaired alveolar epithelial cells by a connexin‐43–dependent mechanism.^89^ This mitochondrial transfer resulted in increased alveolar ATP concentrations in injured mouse epithelial cells. This effect reduced the endotoxin‐induced alveolar injury and increased survival. More recent studies demonstrated that mitochondrial transfer from MSCs to macrophages^90^ occurred partly through tunneling nanotubes and that this transfer could enhance phagocytic activity of macrophages by improving macrophage bioenergetics.^91^ Collectively, mitochondrial transfer from MSCs to alveolar epithelium and macrophages reduces lung injury and leads to an increase in fluid clearance and in phagocytic activity, respectively (Figure 2).

## 10 EXPERIMENTAL AND CLINICAL USE OF MSC FOR ARDS

Because there is no specific therapy for ARDS, managing the clinical symptoms is solely supportive. Patients with moderate‐to‐severe ARDS benefits from protective ventilation and muscle relaxants. As such, alternative therapy regimens that can focus multiple mechanisms of injury, maintain or enhance host defenses to pathogens, and facilitate the lung repair process are a treatment goal. As discussed above, COVID‐19 progression mainly depends on the development of cytokine storm, which is triggered by the activation of macrophages and antigen‐presenting cells, thus alerting lymphocytes to the existence of virus that activates the synthesis of proinflammatory factors. In this regard, because of their potent immunoregulatory features, MSCs could have therapeutic benefits in patients with ARDS, who present hyperactivated T cells manifested by the sudden and extreme increase of several cytokines, which result in cytotoxic effects, mainly on the respiratory system. As indicated above, MSCs exert their constructive effects through the release of paracrine factors and EVs and transfer of mitochondria, all of which have anti‐inflammatory and regenerative effects on injured lung endothelium and alveolar pneumocytes (Figure 2; extensively reviewed by Laffey and Matthay^92^). BM‐MSCs improved repair after ventilator‐induced lung injury, facilitated resolution of inflammation, and restored lung function and structure.^93^ The MSC‐derived EVs or conditioned media from MSCs diminished the injured alveolar epithelial permeability by restoring directional sodium and fluid transport in rats,^94^ restored cytokine‐injured cultured type II alveolar cells by the release of angiopoietin‐1,^95^ and improved alveolar fluid clearance in an ex vivo endotoxin‐injured lung model in humans, partly by the release of keratinocyte growth factor,^96^ potentially having a pivotal role in the resolution of ventilator‐induced lung injury.^92^ The MSC‐conditioned media rescued rats from bleomycin‐induced lung injury by relieving inflammatory markers and apoptosis and by reducing collagen‐rich scar tissue and fibrosis.^97^ MSCs also augmented the resolution of endotoxin‐induced lung injury in mice by the release of a lipid mediator, lipoxin A4.^98^ Additionally, MSC‐derived EVs diminished lung injury, reduced bacterial load, and enhanced survival in bacterial pneumonia in mice^99^ and improved airway compliance in humans.^100^

As IL‐6 secreted by the macrophages is a central player in COVID‐19 and because it is induced by MSC‐derived PGE_2_, the blockage of IL‐6 through PGE_2_ inhibitors may have a suppressive role in cytokine storm in COVID‐19. Indeed, PGE_2_ regulates the production of a wide array of cytokines and upregulates both IL‐10 and IL‐6 in activated macrophages,^101^ which in turn triggers cytopathic effects of cytokine storm. PGE_2_ is also effective in elevating IL‐10 levels only when it is added to cells in which p38 kinase has been activated.^101^ However, the augmentation effect of PGE_2_ on IL‐6 levels is independent of p38 kinase activity, and p38 kinase inhibitors are able to inhibit IL‐6 production in activated macrophages by inhibiting PGE_2_ synthesis. Collectively, MSC‐derived PGE_2_ has a central role in controlling the COVID‐19‐induced IL‐6‐ and IL‐10‐mediated cytokine storm.

MSCs also have the potential of releasing antibacterial factors and therefore stimulating monocyte/macrophage phagocytosis.^96^ The secretion of LL‐37 (β‐cathelicidin), an antimicrobial peptide, contributed to the antibacterial properties of MSCs.^102^ Toll‐like receptors (TLRs) that are activated by viral RNA (TLR3) (as in COVID‐19) and viral unmethylated CpG‐DNA (TLR9) lead to downstream cell signaling pathways and result in MSC activation.^103^ However, mediators responsible for ameliorating respiratory viral‐induced lung injuries remain unclear.^44^ Another an important question that still remains to be resolved is whether MSC act directly against viral pathogens through antiviral proteins such as IFN‐stimulated genes, by stimulating antiviral T‐cell actions, or whether they are due to overall anti‐inflammatory actions.^44^

As mentioned above, transfer of MSC mitochondria may also occur in the alveolar healing mechanism. Experiments in mice showed reduced endotoxin‐induced alveolar damage and increased survival, possibly via a gap junction protein (connexin‐43)‐dependent mechanism that augmented intracellular ATP levels in injured epithelial cells.^89^ More recently, mitochondrial transfer was demonstrated to occur from MSCs to macrophages,^90^ through tunneling nanotubes,^91^ which could enhance macrophage phagocytosis (Figure 2).

Given that MSC‐based interventions in experimental ARDS offers promising results, phase I/II clinical trials have been pursued to assess the efficacy and also safety of human MSC injections in patients with ARDS. A recent literature review by Qu et al^104^ found only nine trials (two were implemented in COVID‐19‐induced ARDS) in which the therapeutic success of MSCs from different sources was examined in 200 patients with ARDS over the last 30 years. All MSCs were allogeneic from bone marrow, umbilical cord stroma or blood, adipose tissue, or menstrual blood. The first study by Zheng et al in 2014 showed that no serious adverse events related to MSC administration were observed in 12 patients with ARDS.^105^ A single delivery of 1 × 10^6^ ADSCs per kilogram of body weight was transplanted intravenously. Length of hospital stay, ventilator‐free days, and ICU‐free days at day 28 after treatment were similar between the cell‐treated and control groups. Serum surfactant protein‐D levels at day 5 were significantly lower than those at day 0 in the MSC group. The authors concluded that administration of allogeneic ADSCs appeared to be safe and feasible in the treatment of ARDS. However, the clinical effect with the doses of MSCs used was weak, and further optimization of this strategy was needed. In a single case report (59‐year‐old man with a severe ARDS), Chang et al^106^ administered umbilical cord blood‐derived MSCs (1 × 10^6^/kg) via intratracheal route; they noted an immediate improvement in his mental status, his lung compliance, and his chest radiography over the course of 3 days. Wilson et al in 2015^107^ published the results of a dose‐escalation trial. Intravenous (IV) administration of allogeneic BM‐MSCs were tolerated well in nine patients with moderate‐to‐severe ARDS (each group consisting of three patients) who received a single delivery of 1, 5, or 10 × 10^6^ cells/kg. No specific therapeutic benefit was reported in this trial. In the same year, Simonson et al administered BM‐MSCs (single dose 2 × 10^6^ cells/kg) to two patients with severe ARDS and found that both patients exhibited improved lung function and survived multiorgan failure.^108^ More recently, the START study by Matthay et al demonstrated that no hemodynamic or respiratory adverse events related to allogeneic BM‐MSC infusion (single dose 10 × 10^6^ cells/kg) were observed over a 60‐day follow‐up period, and although the 28‐day mortality was higher in the MSC group compared with placebo, there was no significant difference between the groups.^109^ More recently, Yip et al^110^ investigated the therapeutic potential of single dose IV infusion of 1, 5, or 10 × 10^6^ UC‐MSCs/kg in nine moderate‐to‐severe cases of ARDS. They noted a trend of improvement in PaO_2_/FiO_2_ and shorter ventilator‐free ICU‐free days. Taken together, these clinical trials revealed improvements in radiographic findings, pulmonary function (lung compliance, tidal volumes, PaO_2_/FiO_2_ ratio, alveolo‐capillary injury), and inflammatory biomarker.^104^ However, there is still an urgent requirement for further assessment of the efficacy of MSCs, especially in COVID‐19‐induced ARDS cases. In addition, low mobilization of MSCs to the sites of injury and poor survival of transplanted MSCs in the harsh microenvironment are obstacles faced by clinical translation.^111^

## 11 CLINICAL USE OF MSCs IN PATIENTS WITH COVID‐19

As indicated above, cytokine storm leads to the advance of pulmonary damage and ARDS in patients with COVID‐19. Previous coronavirus pandemics (SARS and MERS) revealed that the immunosuppressive approach with corticosteroids was not routinely recommended and might exacerbate COVID‐19‐associated lung injury.^112^ IL‐6 blockade may benefit surviving patients with hyperinflammation. In some centers, including in China,^113^ multicenter, randomized controlled trials of tocilizumab (IL‐6 receptor blockade, licensed for cytokine release syndrome) are underway in patients with COVID‐19 pneumonia and elevated IL‐6.

Given that MSC trials were safe and slightly effective in patients with ARDS, at least in some cases and in tested doses, there is a growing demand for the application of MSC‐based interventions in patients with COVID‐19. Patients in the ICU would definitely be the first choice for an MSC‐based trial. As of 5 June 2020, two reports have appeared in the literature addressing the efficacy of completed (up to 2‐4 weeks' follow‐up) MSC transplantations in patients with COVID‐19. The first report came from a group of physicians and scientists from China, who briefly described the clinical outcome of a 65‐year‐old female patient with COVID‐19.^114^ Her oxygen saturation was around 81% the day after hospital admission. According to the guidelines, she was treated with antiviral therapy of lopinavir/ritonavir, IFN‐α inhalation, and oseltamivir and IV injection of moxifloxacin, xuebijing, methylprednisolone, and immunoglobulin. Almost 10 days after admission, she developed severe organ failure. Upon cessation of the glucocorticoid and antiviral therapy, she received a triple‐IV infusion of allogenic UC‐MSCs (5 × 10^6^ cells each time). No obvious adverse effects were observed after injections. After the second administration, the serum bilirubin, CRP, and ALT/AST gradually decreased, along with the improvement of some vital signs. She was extubated, and lung improvement was verified in the chest CT images. Two days after the third infusion, her throat swabs were negative for SARS‐CoV‐2. At day 20 after admission, she was transferred out of the ICU.

The second report was a mini clinical trial from a multidisciplinary, multinational group of scientists and physicians.^115^ The researchers compared seven patients with COVID‐19 pneumonia (one critically severe, four serious, and two common; aged 18‐95 years) who were not responding to standard treatment. They received certified, Good Manufacturing Practices‐grade single dose 1 × 10^6^ UC‐MSC/kg^116^ as opposed to three patients in the control group who received no cells. All seven patients in the treatment group recovered in the 14‐day follow‐up. In the control group, one patient died, and another developed ARDS. Only one patient in the control group was stable. No complications were noted in the treatment group. Within a few days in the treated group, the oxygen saturation and biomarkers for inflammation and tissue injury such as CRP, AST, creatine kinase activity, and myoglobin normalized. Significant improvements were seen in the radiologic signs in the follow‐up CT scans of the lungs. The limitations of this study were the small sample size and short‐term follow‐up as well as the lack of statistical power and randomization.^117^ However, this mini trial also showed that infused MSCs remained negative for angiotensin‐converting enzyme 2, a cell surface receptor that is required for SARS‐CoV‐2 virus to attach to the alveolar epithelial cells,^118^ meaning that transplanted MSCs did not differentiate and remained free of virus.^119^

In an extremely short time interval, as of 5 June 2020, 36 MSC trials for COVID‐19 were registered to ClinicalTrials.gov, primarily aiming to rescue patients with severe or critical COVID‐19 (Table 2). Apparently, the number of trials will rise, because we are aware that in the corresponding author's country of residence, two phase II/III trials (enrolling 10 and 100 patients) and 25 single cases were recently approved by the Stem Cell Advisory and Review Board of the Ministry of Health (unpublished data). In the United States, the Food and Drug Administration approved stem cell therapy for COVID‐19 followed by the announcement of immediate actions of many cell manufacturing and treatment companies, some of which also publicized the patient recruitment. Although the full evidence for the success of MSC treatment in patients with COVID‐19 is still unavailable, we think that MSCs have a place in treatment and improvement for thousands of hopeless patients around the world in this widest pandemic to have ever happened in modern times. More recently, the American Academy of Stem Cell Physicians has announced recommendations for the treatment of COVID‐19, which it has sent to the director and representatives of the World Health Organization.^120^ Although the source of cells is different than MSCs, the treatment plan recommends the administration of umbilical cord blood‐derived mononuclear cells (1 × 10^6^ cells/kg) daily for hospitalized patients. Beside the aforementioned potential benefits of MSCs, it is also very important to point out that there is a good deal of concern about clinics offering unproven stem cell treatments for COVID‐19^121^; as such, reviewers and oversight bodies will be looking for a systematic appraisal of evidence and risk‐benefit analysis of current trials.

**TABLE 2:** MSC‐based clinical trials registered to www.ClinicalTrials.gov database in COVID‐19 as of 6 June 2020

ClinicalTrials identifier	Study design	Current status	Study location	MSC type	Estimated enrollment/route of delivery	Primary outcome
NCT04348461	Phase II	Not yet recruiting	Spain	ADSC	100/IV	Survival rate Safety assessed by adverse event rate
NCT04366323	Phase I/II	Not yet recruiting	Spain	ADSC	26/IV	Safety assessed by adverse event rate Survival rate
NCT04352803	Phase I	Not yet recruiting	United States	ADSC	20/IV	Safety—Incidence of unexpected adverse events
NCT04362189	Phase II	Not yet recruiting	United States	ADSC	100/IV	Change from baseline in levels of d‐dimer, interleukin‐6, C‐reactive protein, OI, PCR test SARS‐CoV‐2
NCT04348435	Phase II	Enrolling by invitation	United States	ADSC	100/IV	Incidence of hospitalization Incidence of associated symptoms
NCT04346368	Phase I/II	Not yet recruiting	China	BM‐MSC	20/IV	Changes of oxygenation index (PaO_2_/FiO_2_) Side effects
NCT04377334	Phase II	Not yet recruiting	Germany	BM‐MSC	40/IV	Improvement of lung injury score, 0‐16 points, severity increasing with higher points
NCT04397796	Phase I	Not yet recruiting	United States	BM‐MSC	45/NA	Incidence of adverse effects, mortality, and cause of death within 30 days of randomization Number of ventilator‐free days within 60 days of randomization
NCT04400032	Phase I	Not yet recruiting	Canada	BM‐MSC	9/IV	Number of participants alive by day 28 Number of participants with ventilator‐free days by day 28
NCT04336254	Phase I/II	Recruiting	China	DP‐MSC	20/IV	TTCI Recovery of lung lesion Immune function
NCT04302519	Early phase I	Not yet recruiting	China	DP‐MSC	24/IV	Time to disappearance of ground‐glass shadow in the lungs
NCT04276987	phase I	Not yet recruiting	China	MSC‐derived exosomes	30/aerosol inhalation	Adverse effects and severe adverse reactions TTCI
NCT04315987	Phase II	Not yet recruiting	Brazil	NestaCell	90/IV	Time to disappearance of ground‐glass shadow in the lungs
NCT04389450	Phase II	Not yet recruiting	United States	PLX‐PAD MSC	140/IM	Number of ventilator‐free days by day 28
NCT04339660	Phase I/II	Recruiting	China	UC‐MSC	30/IV	The immune function Increase in blood oxygen saturation
NCT04273646	NA	Not yet recruiting	China	UC‐MSC	48/IV	Pneumonia severity index Oxygenation index (PaO_2_/FiO_2_)
NCT04269525	Phase II	Recruiting	China	UC‐MSC	10/IV	Oxygenation index (PaO_2_/FiO_2_)
NCT04333368	Phase I/II	Recruiting	France	UC‐MSC	40/IV	Respiratory efficacy evaluated by the increase in PaO_2_/FiO_2_
NCT03042143	Phase I/II	Recruiting	United Kingdom	UC‐MSC (CD362 enriched)	75/NA	OI Incidence of serious adverse events
NCT04355728	Phase I/II	Recruiting	United States	UC‐MSC	24/IV	Incidence of prespecified infusion‐associated adverse events Incidence of severe adverse events
NCT04366271	Phase II	Recruiting	Spain	UC‐MSC	106/IV	Mortality due to lung involvement at 28 days of treatment
NCT04398303	Phase I/II	Not yet recruiting	United States	UC‐MSC	70/IV	Mortality at day 30
NCT04313322	Phase I	Recruiting	Jordan	WJ‐MSC	5/IV	Clinical improvement Improvement in CT Scan
NCT04390152	Phase I/II	Not yet recruiting	Colombia	WJ‐MSC	40/IV	Evaluation of efficacy of cells defined by mortality at 28 days of application
NCT04390139	Phase I/II	Recruiting	Spain	WJ‐MSC	30/EV	All‐cause mortality at day 28, number of patients who died
NCT04382547	Phase I/II	Enrolling by invitation	Belarus	OMD‐MSC	40/NA	Number of cured patients Number of patients with treatment‐related adverse events
NCT04349631	Phase II	Enrolling by invitation	United States	ADSC	56/IV	Incidence of hospitalization Incidence of associated symptoms
NCT04416139	Phase II	Recruiting	Mexico	NR	10/IV	PaO_2_/FiO_2_ ratio Heart rate per minute Respiratory rate per minute Changes in body temperature
NCT04371601	Early phase I	Active, not recruiting	China	NR	60/NA	Changes of oxygenation index (PaO_2_/FiO_2_), blood gas test
NCT04345601	Early phase I	Not yet recruiting	United States	NR	30/IV	Incidence of unexpected adverse events Improved oxygen saturations ≥93%
NCT04288102	Phase II	Recruiting	China	NR	90/IV	Size of lesion area and severity of pulmonary fibrosis by chest CT
NCT04252118	Phase I	Recruiting	China	NR	20/IV	Size of lesion area by chest radiography or CT Side effects in the MSC treatment group
NCT04366063	Phase II/III	Recruiting	Iran	NR	60/IV	Adverse events assessment Blood oxygen saturation
NCT04392778	Phase I/II	Recruiting	Turkey	NR	30/NA	Improvement of clinical symptoms
NCT04361942	Phase II	Recruiting	Spain	NR	24/IV	Proportion of patients who have achieved clinical response Proportion of patients who have achieved radiological responses
NCT04371393	Phase III	Recruiting	United States	NR	300/IV	Number of all‐cause mortalities within 30 days of randomization

Abbreviations: ADSC, adipose tissue–derived stem cell; BM‐MSC, bone marrow mesenchymal stem cell; CT, computed tomography; DP‐MSC, dental pulp mesenchymal stem cell; EV, extracellular vesicle; FiO_2_, fraction of inspired oxygen; IV, intravenous; MSC, mesenchymal stem cell; NA, not available; NR, not reported; OI, oxygenation index; OMD‐MSC, olfactory mucosa‐derived MSC; PaO_2_, partial pressure of oxygen; PCR, polymerase chain reaction; PLX‐PAD, placental mesenchymal‐like adherent stromal cells; SARS‐CoV‐2, severe acute respiratory syndrome coronavirus 2; TTCI, time to clinical improvement; UC‐MSC, umbilical cord mesenchymal stem cell; WJ‐MSC, Wharton's jelly mesenchymal stem cell.

## 12 CONCLUSION

Besides the central question regarding whether MSC therapy would be a proven stem cell therapy for patients with COVID‐19, obviously, there are still many unanswered critical questions, such as (a) which source of MSCs would be more beneficial; (b) with what cell dose and how many times should MSCs be administered; (c) what is the best route for the cell administration (IV or intratracheal/intrabronchial); (d) what is the best time to intervene with MSCs in patients with COVID‐19; (e) what would be the optimal follow‐up time for cell‐treated patients to confirm the efficacy of a given therapy schema; and (f) is it beneficial to add any drugs to enhance or support the MSC treatment? The answers to some of the above questions largely remain obscured by the lack of comparison studies. Regarding the route of cell administration, the IV route seems to be the best choice for greater cell survival, practicability of bedside application, and likelihood of paracrine effect exertion. On the other hand, one of the main problems of the MSC‐based therapeutic interventions is that high expectations based on the strong therapeutic effect in preclinical models do not match up with the reality of low efficacy of MSCs in clinical trials. One explanation of this might be that real human subjects (patients) present with much more heterogeneous disease states and varying conditions than inbred animal strains; therefore, we need much better understanding of the patient populations, aiming MSC therapy at those who will be most likely to respond. This problem is crucially relevant to patients with COVID‐19‐induced ARDS.

## CONFLICT OF INTEREST

The authors declared no potential conflicts of interest.

## AUTHOR CONTRIBUTIONS

A.C.: conception and design, manuscript writing; H.C.: manuscript writing.

## Data Availability

Data sharing is not applicable to this article as no new data were created or analyzed in this study.

## References

[1] Su S , Wong G , Shi W , et al. Epidemiology, genetic recombination, and pathogenesis of coronaviruses. Trends Microbiol. 2016;24:490‐502. 27012512 10.1016/j.tim.2016.03.003 PMC7125511

[2] WHO Coronavirus Disease (COVID‐19) Dashboard. World Health Organization Web site. https://covid19.who.int/. Accessed June 23, 2020.

[3] COVID‐19 Coronavirus Pandemic. Worldometer.info Web site. https://www.worldometers.info/coronavirus/. Accessed June 23, 2020.

[4] Wu Z , McGoogan JM . Characteristics of and important lessons from the coronavirus disease 2019 (COVID‐19) outbreak in China: summary of a report of 72314 cases from the Chinese Center for Disease Control and Prevention. JAMA. 2020;323:1239. 32091533 10.1001/jama.2020.2648

[5] Dong Y , Mo X , Hu Y , et al. Epidemiology of COVID‐19 among children in China. Pediatrics. 2020;145:e20200702. 32179660 10.1542/peds.2020-0702

[6] Zhou M , Zhang X , Qu J . Coronavirus disease 2019 (COVID‐19): a clinical update. Front Med. 2020;14:126‐135. 32240462 10.1007/s11684-020-0767-8 PMC7115348

[7] Li Q , Guan X , Wu P , et al. Early transmission dynamics in Wuhan, China, of novel coronavirus‐infected pneumonia. N Engl J Med. 2020;382:1199‐1207. 31995857 10.1056/NEJMoa2001316 PMC7121484

[8] Huang C , Wang Y , Li X , et al. Clinical features of patients infected with 2019 novel coronavirus in Wuhan, China. Lancet. 2020;395:497‐506. 31986264 10.1016/S0140-6736(20)30183-5 PMC7159299

[9] Li K , Fang Y , Li W , et al. CT image visual quantitative evaluation and clinical classification of coronavirus disease (COVID‐19). Eur Radiol. 2020;382:1199‐1207. 10.1007/s00330-020-06817-6 PMC7095246 32215691

[10] Lu H . Drug treatment options for the 2019‐new coronavirus (2019‐nCoV). Biosci Trends. 2020;14:69‐71. 31996494 10.5582/bst.2020.01020

[11] Valk SJ , Piechotta V , Chai KL , et al. Convalescent plasma or hyperimmune immunoglobulin for people with COVID‐19: a rapid review. Cochrane Database Syst Rev. 2020;(5):CD013600. 32406927 10.1002/14651858.CD013600 PMC7271896

[12] Wang W , Xu Y , Gao R , et al. Detection of SARS‐CoV‐2 in different types of clinical specimens. JAMA. 2020;323:1843‐1844. 32159775 10.1001/jama.2020.3786 PMC7066521

[13] Dhama K , Patel SK , Pathak M , et al. An update on SARS‐CoV‐2/COVID‐19 with particular reference to its clinical pathology, pathogenesis, immunopathology and mitigation strategies. Travel Med Infect Dis. 2020;101755. 32479816 10.1016/j.tmaid.2020.101755 PMC7260597

[14] Singhal T . A review of coronavirus disease‐2019 (COVID‐19). Indian J Pediatr. 2020;87:281‐286. 32166607 10.1007/s12098-020-03263-6 PMC7090728

[15] Chen N , Zhou M , Dong X , et al. Epidemiological and clinical characteristics of 99 cases of 2019 novel coronavirus pneumonia in Wuhan, China: a descriptive study. Lancet. 2020;395:507‐513. 32007143 10.1016/S0140-6736(20)30211-7 PMC7135076

[16] Huang P , Liu T , Huang L , et al. Use of chest CT in combination with negative RT‐PCR assay for the 2019 novel coronavirus but high clinical suspicion. Radiology. 2020;295:22‐23. 32049600 10.1148/radiol.2020200330 PMC7233360

[17] Wu D , Yang XO . TH17 responses in cytokine storm of COVID‐19: an emerging target of JAK2 inhibitor fedratinib. J Microbiol Immunol Infect. 2020;53:368‐370. 32205092 10.1016/j.jmii.2020.03.005 PMC7156211

[18] Huang KJ , Su IJ , Theron M , et al. An interferon‐gamma‐related cytokine storm in SARS patients. J Med Virol. 2005;75:185‐194. 15602737 10.1002/jmv.20255 PMC7166886

[19] Behrens EM , Koretzky GA . Review: cytokine storm syndrome: looking toward the precision medicine era. Arthritis Rheumatol. 2017;69:1135‐1143. 28217930 10.1002/art.40071

[20] Tisoncik JR , Korth MJ , Simmons CP , Farrar J , Martin TR , Katze MG . Into the eye of the cytokine storm. Microbiol Mol Biol Rev. 2012;76:16‐32. 22390970 10.1128/MMBR.05015-11 PMC3294426

[21] Ramos‐Casals M , Brito‐Zerón P , López‐Guillermo A , Khamashta MA , Bosch X . Adult haemophagocytic syndrome. Lancet. 2014;383:1503‐1516. 24290661 10.1016/S0140-6736(13)61048-X

[22] Ruan Q , Yang K , Wang W , et al. Clinical predictors of mortality due to COVID‐19 based on an analysis of data of 150 patients from Wuhan, China. Intensive Care Med. 2020;46:846‐848. 32125452 10.1007/s00134-020-05991-x PMC7080116

[23] Mehta P , McAuley DF , Brown M , et al. COVID‐19: consider cytokine storm syndromes and immunosuppression. Lancet. 2020;395:1033‐1034. 32192578 10.1016/S0140-6736(20)30628-0 PMC7270045

[24] Wong CK , Lam CW , Wu AK , et al. Plasma inflammatory cytokines and chemokines in severe acute respiratory syndrome. Clin Exp Immunol. 2004;136:95‐103. 15030519 10.1111/j.1365-2249.2004.02415.x PMC1808997

[25] Mahallawi WH , Khabour OF , Zhang Q , et al. MERS‐CoV infection in humans is associated with a pro‐inflammatory Th1 and Th17 cytokine profile. Cytokine. 2018;104:8‐13. 29414327 10.1016/j.cyto.2018.01.025 PMC7129230

[26] Varga Z , Flammer AJ , Steiger P , et al. Endothelial cell infection and endotheliitis in COVID‐19. Lancet. 2020;395:1417‐1418. 32325026 10.1016/S0140-6736(20)30937-5 PMC7172722

[27] Leisman DE , Deutschman CS , Legrand M . Facing COVID‐19 in the ICU: vascular dysfunction, thrombosis, and dysregulated inflammation. Intensive Care Med. 2020;46:1105‐1108. 32347323 10.1007/s00134-020-06059-6 PMC7186535

[28] Angstwurm MW , Reininger AJ , Spannagl M . D‐dimer as marker for microcirculatory failure: correlation with LOD and APACHE II scores. Thromb Res. 2004;113:353‐359. 15226089 10.1016/j.thromres.2004.03.013

[29] Bernard GR , Artigas A . The definition of ARDS revisited: 20 years later. Intensive Care Med. 2016;42:640‐642. 26942447 10.1007/s00134-016-4281-z

[30] Guan WJ , Ni ZY , Hu Y , et al. Clinical characteristics of coronavirus disease 2019 in China. N Engl J Med. 2020;382:1708‐1720. 32109013 10.1056/NEJMoa2002032 PMC7092819

[31] Matthay MA , Ware LB , Zimmerman GA . The acute respiratory distress syndrome. J Clin Invest. 2012;122:2731‐2740. 22850883 10.1172/JCI60331 PMC3408735

[32] Ding Y , Wang H , Shen H , et al. The clinical pathology of severe acute respiratory syndrome (SARS): a report from China. J Pathol. 2003;200:282‐289. 12845623 10.1002/path.1440 PMC7168017

[33] Lang ZW , Zhang LJ , Zhang SJ , et al. A clinicopathological study of three cases of severe acute respiratory syndrome (SARS). Pathology. 2003;35:526‐531. 14660106 10.1080/00313020310001619118 PMC7131316

[34] Xu Z , Shi L , Wang Y , et al. Pathological findings of COVID‐19 associated with acute respiratory distress syndrome. Lancet Respir Med. 2020;8:420‐422. 32085846 10.1016/S2213-2600(20)30076-X PMC7164771

[35] Pittenger MF , Mackay AM , Beck SC , et al. Multilineage potential of adult human mesenchymal stem cells. Science. 1999;284:143‐147. 10102814 10.1126/science.284.5411.143

[36] Dominici M , Le Blanc K , Mueller I , et al. Minimal criteria for defining multipotent mesenchymal stromal cells. The International Society for Cellular Therapy position statement. Cytotherapy. 2006;8:315‐317. 16923606 10.1080/14653240600855905

[37] Bianco P , Cao X , Frenette PS , et al. The meaning, the sense and the significance: translating the science of mesenchymal stem cells into medicine. Nat Med. 2013;19:35‐42. 23296015 10.1038/nm.3028 PMC3998103

[38] Le Blanc K , Davies LC . Mesenchymal stromal cells and the innate immune response. Immunol Lett. 2015;168:140‐146. 25982165 10.1016/j.imlet.2015.05.004

[39] Wang Y , Chen X , Cao W , Shi Y . Plasticity of mesenchymal stem cells in immunomodulation: pathological and therapeutic implications. Nat Immunol. 2014;15:1009‐1016. 25329189 10.1038/ni.3002

[40] Haynesworth SE , Baber MA , Caplan AI . Cytokine expression by human marrow‐derived mesenchymal progenitor cells in vitro: effects of dexamethasone and IL‐1 alpha. J Cell Physiol. 1996;166:585‐592. 8600162 10.1002/(SICI)1097-4652(199603)166:3<585::AID-JCP13>3.0.CO;2-6

[41] Lazarus HM , Haynesworth SE , Gerson SL , Rosenthal NS , Caplan AI . Ex vivo expansion and subsequent infusion of human bone marrow‐derived stromal progenitor cells (mesenchymal progenitor cells): implications for therapeutic use. Bone Marrow Transplant. 1995;16:557‐564. 8528172

[42] Hoogduijn MJ , Lombardo E . Mesenchymal stromal cells anno 2019: dawn of the therapeutic era? Concise review. Stem Cells Translational Medicine. 2019;8:1126‐1134. 31282113 10.1002/sctm.19-0073 PMC6811696

[43] Couto PS , Shatirishvili G , Bersenev A , Verter F . First decade of clinical trials and published studies with mesenchymal stromal cells from umbilical cord tissue. Regen Med. 2019;14:309‐319. 31070115 10.2217/rme-2018-0171

[44] Khoury M , Cuenca J , Cruz FF , Figueroa FE , Rocco PRM , Weiss DJ . Current status of cell‐based therapies for respiratory virus infections: applicability to COVID‐19. Eur Respir J. 2020;55:2000858. 32265310 10.1183/13993003.00858-2020 PMC7144273

[45] Frieman MB , Chen J , Morrison TE , et al. SARS‐CoV pathogenesis is regulated by a STAT1 dependent but a type I, II and III interferon receptor independent mechanism. PLoS Pathog. 2010;6:e1000849. 20386712 10.1371/journal.ppat.1000849 PMC2851658

[46] Gregory CA , Prockop DJ , Spees JL . Non‐hematopoietic bone marrow stem cells: molecular control of expansion and differentiation. Exp Cell Res. 2005;306:330‐335. 15925588 10.1016/j.yexcr.2005.03.018

[47] Pontikoglou C , Deschaseaux F , Sensebe L , et al. Bone marrow mesenchymal stem cells: biological properties and their role in hematopoiesis and hematopoietic stem cell transplantation. Stem Cell Rev Rep. 2011;7:569‐589. 21249477 10.1007/s12015-011-9228-8

[48] Aggarwal S , Pittenger MF . Human mesenchymal stem cells modulate allogeneic immune cell responses. Blood. 2005;105:1815‐1822. 15494428 10.1182/blood-2004-04-1559

[49] Karahuseyinoglu S , Cinar O , Kilic E , et al. Biology of stem cells in human umbilical cord stroma: in situ and in vitro surveys. Stem Cells. 2007;25:319‐331. 17053211 10.1634/stemcells.2006-0286

[50] Can A , Balci D . Isolation, culture, and characterization of human umbilical cord stroma‐derived mesenchymal stem cells. Methods Mol Biol. 2011;698:51‐62. 21431510 10.1007/978-1-60761-999-4_5

[51] Coskun H , Can A . The assessment of the in vivo to in vitro cellular transition of human umbilical cord multipotent stromal cells. Placenta. 2015;36:232‐239. 25524058 10.1016/j.placenta.2014.11.024

[52] Horwitz EM , Le Blanc K , Dominici M , et al. Clarification of the nomenclature for MSC: the International Society for Cellular Therapy position statement. Cytotherapy. 2005;7:393‐395. 16236628 10.1080/14653240500319234

[53] He H , Nagamura‐Inoue T , Takahashi A , et al. Immunosuppressive properties of Wharton's jelly‐derived mesenchymal stromal cells in vitro. Int J Hematol. 2015;102:368‐378. 26228529 10.1007/s12185-015-1844-7

[54] Liu X , Feng T , Gong T , et al. Human umbilical cord mesenchymal stem cells inhibit the function of allogeneic activated Vγ9Vδ2 T lymphocytes in vitro. Biomed Res Int. 2015;2015:317801. 25984529 10.1155/2015/317801 PMC4423519

[55] Weiss ML , Anderson C , Medicetty S , et al. Immune properties of human umbilical cord Wharton's jelly‐derived cells. Stem Cells. 2008;26:2865‐2874. 18703664 10.1634/stemcells.2007-1028

[56] Can A , Yigman Z . Mesenchymal stem/stromal cells from neonatal tissues. In: Bolontrade MF , Garcia MG , eds. Mesenchymal Stromal Cells as Tumor Stromal Modulators. New York, NY: Academic Press; 2016:65‐101.

[57] Can A , Celikkan FT , Cinar O . Umbilical cord mesenchymal stromal cell transplantations: a systemic analysis of clinical trials. Cytotherapy. 2017;19:1351‐1382. 28964742 10.1016/j.jcyt.2017.08.004

[58] Atluri S , Manchikanti L , Hirsch JA . Expanded umbilical cord mesenchymal stem cells (UC‐MSCs) as a therapeutic strategy in managing critically ill COVID‐19 patients: the case for compassionate use. Pain Physician. 2020;23:e71‐e83. 32214286

[59] Chu DT , Nguyen Thi Phuong T , Tien NLB , et al. Adipose tissue stem cells for therapy: an update on the progress of isolation, culture, storage, and clinical application. J Clin Med. 2019;8:917. 10.3390/jcm8070917 PMC6678927 31247996

[60] Bourin P , Bunnell BA , Casteilla L , et al. Stromal cells from the adipose tissue‐derived stromal vascular fraction and culture expanded adipose tissue‐derived stromal/stem cells: a joint statement of the International Federation for Adipose Therapeutics and Science (IFATS) and the International Society for Cellular Therapy (ISCT). Cytotherapy. 2013;15:641‐648. 23570660 10.1016/j.jcyt.2013.02.006 PMC3979435

[61] Park MJ , Kwok SK , Lee SH , Kim EK , Park SH , Cho ML . Adipose tissue‐derived mesenchymal stem cells induce expansion of interleukin‐10‐producing regulatory B cells and ameliorate autoimmunity in a murine model of systemic lupus erythematosus. Cell Transplant. 2015;24:2367‐2377. 25506685 10.3727/096368914X685645

[62] Yanez R , Lamana ML , Garcia‐Castro J , et al. Adipose tissue‐derived mesenchymal stem cells have in vivo immunosuppressive properties applicable for the control of the graft‐versus‐host disease. Stem Cells. 2006;24:2582‐2591. 16873762 10.1634/stemcells.2006-0228

[63] Gronthos S , Mankani M , Brahim J , Robey PG , Shi S . Postnatal human dental pulp stem cells (DPSCs) in vitro and in vivo. Proc Natl Acad Sci U S A. 2000;97:13625‐13630. 11087820 10.1073/pnas.240309797 PMC17626

[64] Andrukhov O , Behm C , Blufstein A , Rausch‐Fan X . Immunomodulatory properties of dental tissue‐derived mesenchymal stem cells: implication in disease and tissue regeneration. World J Stem Cells. 2019;11:604‐617. 31616538 10.4252/wjsc.v11.i9.604 PMC6789188

[65] Liu L , Wei X , Ling J , Wu L , Xiao Y . Expression pattern of Oct‐4, Sox2, and c‐Myc in the primary culture of human dental pulp derived cells. J Endod. 2011;37:466‐472. 21419292 10.1016/j.joen.2010.12.012

[66] Pierdomenico L , Bonsi L , Calvitti M , et al. Multipotent mesenchymal stem cells with immunosuppressive activity can be easily isolated from dental pulp. Transplantation. 2005;80:836‐842. 16210973 10.1097/01.tp.0000173794.72151.88

[67] Yildirim S , Zibandeh N , Genc D , et al. The comparison of the immunologic properties of stem cells isolated from human exfoliated deciduous teeth, dental pulp, and dental follicles. Stem Cells Int. 2016;2016:4682875. 26770205 10.1155/2016/4682875 PMC4684887

[68] Shi Y , Wang Y , Li Q , et al. Immunoregulatory mechanisms of mesenchymal stem and stromal cells in inflammatory diseases. Nat Rev Nephrol. 2018;14:493‐507. 29895977 10.1038/s41581-018-0023-5

[69] Schraufstatter IU , Discipio RG , Zhao M , et al. C3a and C5a are chemotactic factors for human mesenchymal stem cells, which cause prolonged ERK1/2 phosphorylation. J Immunol. 2009;182:3827‐3836. 19265162 10.4049/jimmunol.0803055

[70] Vasandan AB , Jahnavi S , Shashank C , Prasad P , Kumar A , Prasanna SJ . Human mesenchymal stem cells program macrophage plasticity by altering their metabolic status via a PGE(2)‐dependent mechanism. Sci Rep. 2016;6:38308. 27910911 10.1038/srep38308 PMC5133610

[71] Mittal M , Tiruppathi C , Nepal S , et al. TNFα‐stimulated gene‐6 (TSG6) activates macrophage phenotype transition to prevent inflammatory lung injury. Proc Natl Acad Sci U S A. 2016;113:e8151‐e8158. 27911817 10.1073/pnas.1614935113 PMC5167170

[72] Wang G , Cao K , Liu K , et al. Kynurenic acid, an IDO metabolite, controls TSG‐6‐mediated immunosuppression of human mesenchymal stem cells. Cell Death Differ. 2018;25:1209‐1223. 29238069 10.1038/s41418-017-0006-2 PMC6030103

[73] Zhou BO , Yue R , Murphy MM , Peyer JG , Morrison SJ . Leptin‐receptor‐expressing mesenchymal stromal cells represent the main source of bone formed by adult bone marrow. Cell Stem Cell. 2014;15:154‐168. 24953181 10.1016/j.stem.2014.06.008 PMC4127103

[74] Molina EJ , Palma J , Gupta D , et al. Reverse remodeling is associated with changes in extracellular matrix proteases and tissue inhibitors after mesenchymal stem cell (MSC) treatment of pressure overload hypertrophy. J Tissue Eng Regen Med. 2009;3:85‐91. 19065545 10.1002/term.137

[75] Yan X , Liu Y , Han Q , et al. Injured microenvironment directly guides the differentiation of engrafted Flk‐1(+) mesenchymal stem cell in lung. Exp Hematol. 2007;35:1466‐1475. 17637496 10.1016/j.exphem.2007.05.012

[76] Zhang C , Zhu Y , Zhang Y , Gao L , Zhang N , Feng H . Therapeutic potential of umbilical cord mesenchymal stem cells for inhibiting myofibroblastic differentiation of irradiated human lung fibroblasts. Tohoku J Exp Med. 2015;236:209‐217. 26105694 10.1620/tjem.236.209

[77] Zhang B , Yin Y , Lai RC , Tan SS , Choo ABH , Lim SK . Mesenchymal stem cells secrete immunologically active exosomes. Stem Cells Dev. 2014;23:1233‐1244. 24367916 10.1089/scd.2013.0479

[78] Raposo G , Stoorvogel W . Extracellular vesicles: exosomes, microvesicles, and friends. J Cell Biol. 2013;200:373‐383. 23420871 10.1083/jcb.201211138 PMC3575529

[79] Allan D , Tieu A , Lalu M , Burger D . Mesenchymal stromal cell‐derived extracellular vesicles for regenerative therapy and immune modulation: progress and challenges toward clinical application. Stem Cells Translational Medicine. 2020;9:39‐46. 31411820 10.1002/sctm.19-0114 PMC6954691

[80] Abels ER , Breakefield XO . Introduction to extracellular vesicles: biogenesis, RNA cargo selection, content, release, and uptake. Cell Mol Neurobiol. 2016;36:301‐312. 27053351 10.1007/s10571-016-0366-z PMC5546313

[81] De Jong OG , Van Balkom BW , Schiffelers RM , et al. Extracellular vesicles: potential roles in regenerative medicine. Front Immunol. 2014;5:608. 25520717 10.3389/fimmu.2014.00608 PMC4253973

[82] Bruno S , Deregibus MC , Camussi G . The secretome of mesenchymal stromal cells: role of extracellular vesicles in immunomodulation. Immunol Lett. 2015;168:154‐158. 26086886 10.1016/j.imlet.2015.06.007

[83] Najar M , Raicevic G , Fayyad‐Kazan H , Bron D , Toungouz M , Lagneaux L . Mesenchymal stromal cells and immunomodulation: a gathering of regulatory immune cells. Cytotherapy. 2016;18:160‐171. 26794710 10.1016/j.jcyt.2015.10.011

[84] Cha JM , Shin EK , Sung JH , et al. Efficient scalable production of therapeutic microvesicles derived from human mesenchymal stem cells. Sci Rep. 2018;8:1171. 29352188 10.1038/s41598-018-19211-6 PMC5775399

[85] Lelek J , Zuba‐Surma EK . Perspectives for future use of extracellular vesicles from umbilical cord‐ and adipose tissue‐derived mesenchymal stem/stromal cells in regenerative therapies‐synthetic review. Int J Mol Sci. 2020;21:1–19. 10.3390/ijms21030799 PMC7036930 31991836

[86] Curley GF , Hayes M , Ansari B , et al. Mesenchymal stem cells enhance recovery and repair following ventilator‐induced lung injury in the rat. Thorax. 2012;67:496‐501. 22106021 10.1136/thoraxjnl-2011-201059

[87] Park J , Kim S , Lim H , et al. Therapeutic effects of human mesenchymal stem cell microvesicles in an ex vivo perfused human lung injured with severe *E. coli* pneumonia. Thorax. 2019;74:43‐50. 30076187 10.1136/thoraxjnl-2018-211576 PMC6295323

[88] McAuley DF , Curley GF , Hamid UI , et al. Clinical grade allogeneic human mesenchymal stem cells restore alveolar fluid clearance in human lungs rejected for transplantation. Am J Physiol Lung Cell Mol Physiol. 2014;306:L809‐L815. 24532289 10.1152/ajplung.00358.2013 PMC4010648

[89] Islam MN , Das SR , Emin MT , et al. Mitochondrial transfer from bone‐marrow‐derived stromal cells to pulmonary alveoli protects against acute lung injury. Nat Med. 2012;18:759‐765. 22504485 10.1038/nm.2736 PMC3727429

[90] Phinney DG , Di Giuseppe M , Njah J , et al. Mesenchymal stem cells use extracellular vesicles to outsource mitophagy and shuttle microRNAs. Nat Commun. 2015;6:8472. 26442449 10.1038/ncomms9472 PMC4598952

[91] Jackson MV , Morrison TJ , Doherty DF , et al. Mitochondrial transfer via tunneling nanotubes is an important mechanism by which mesenchymal stem cells enhance macrophage phagocytosis in the in vitro and in vivo models of ARDS. Stem Cells. 2016;34:2210‐2223. 27059413 10.1002/stem.2372 PMC4982045

[92] Laffey JG , Matthay MA . Fifty years of research in ARDS. Cell‐based therapy for acute respiratory distress syndrome. Biology and potential therapeutic value. Am J Respir Crit Care Med. 2017;196:266‐273. 28306336 10.1164/rccm.201701-0107CP PMC5549868

[93] Hayes M , Masterson C , Devaney J , et al. Therapeutic efficacy of human mesenchymal stromal cells in the repair of established ventilator‐induced lung injury in the rat. Anesthesiology. 2015;122:363‐373. 25490744 10.1097/ALN.0000000000000545

[94] Goolaerts A , Pellan‐Randrianarison N , Larghero J , et al. Conditioned media from mesenchymal stromal cells restore sodium transport and preserve epithelial permeability in an in vitro model of acute alveolar injury. Am J Physiol Lung Cell Mol Physiol. 2014;306:L975‐L985. 24682451 10.1152/ajplung.00242.2013 PMC4042188

[95] Fang X , Neyrinck AP , Matthay MA , Lee JW . Allogeneic human mesenchymal stem cells restore epithelial protein permeability in cultured human alveolar type II cells by secretion of angiopoietin‐1. J Biol Chem. 2010;285:26211‐26222. 20554518 10.1074/jbc.M110.119917 PMC2924032

[96] Lee JW , Krasnodembskaya A , McKenna DH , et al. Therapeutic effects of human mesenchymal stem cells in ex vivo human lungs injured with live bacteria. Am J Respir Crit Care Med. 2013;187:751‐760. 23292883 10.1164/rccm.201206-0990OC PMC3678109

[97] Shen Q , Chen B , Xiao Z , et al. Paracrine factors from mesenchymal stem cells attenuate epithelial injury and lung fibrosis. Mol Med Rep. 2015;11:2831‐2837. 25514921 10.3892/mmr.2014.3092

[98] Fang X , Abbott J , Cheng L , et al. Human mesenchymal stem (stromal) cells promote the resolution of acute lung injury in part through lipoxin A4. J Immunol. 2015;195:875‐881. 26116507 10.4049/jimmunol.1500244

[99] Monsel A , Zhu YG , Gennai S , et al. Therapeutic effects of human mesenchymal stem cell‐derived microvesicles in severe pneumonia in mice. Am J Respir Crit Care Med. 2015;192:324‐336. 26067592 10.1164/rccm.201410-1765OC PMC4584251

[100] Gennai S , Monsel A , Hao Q , Park J , Matthay MA , Lee JW . Microvesicles derived from human mesenchymal stem cells restore alveolar fluid clearance in human lungs rejected for transplantation. Am J Transplant. 2015;15:2404‐2412. 25847030 10.1111/ajt.13271 PMC4792255

[101] Williams JA , Pontzer CH , Shacter E . Regulation of macrophage interleukin‐6 (IL‐6) and IL‐10 expression by prostaglandin E2: the role of p38 mitogen‐activated protein kinase. J Interferon Cytokine Res. 2000;20:291‐298. 10762076 10.1089/107999000312423

[102] Krasnodembskaya A , Song Y , Fang X , et al. Antibacterial effect of human mesenchymal stem cells is mediated in part from secretion of the antimicrobial peptide LL‐37. Stem Cells. 2010;28:2229‐2238. 20945332 10.1002/stem.544 PMC3293245

[103] Waterman RS , Tomchuck SL , Henkle SL , Betancourt AM . A new mesenchymal stem cell (MSC) paradigm: polarization into a pro‐inflammatory MSC1 or an immunosuppressive MSC2 phenotype. PLoS One. 2010;5:e10088. 20436665 10.1371/journal.pone.0010088 PMC2859930

[104] Qu W , Wang Z , Hare JM , et al. Cell‐based therapy to reduce mortality from COVID‐19: systematic review and meta‐analysis of human studies on acute respiratory distress syndrome. Stem Cells Translational Medicine. 2020;9. 10.1002/sctm.20-0146 PMC7300743 32472653

[105] Zheng G , Huang L , Tong H , et al. Treatment of acute respiratory distress syndrome with allogeneic adipose‐derived mesenchymal stem cells: a randomized, placebo‐controlled pilot study. Respir Res. 2014;15:39. 24708472 10.1186/1465-9921-15-39 PMC3994204

[106] Chang Y , Park SH , Huh JW , et al. Intratracheal administration of umbilical cord blood‐derived mesenchymal stem cells in a patient with acute respiratory distress syndrome. J Korean Med Sci. 2014;29:438‐440. 24616596 10.3346/jkms.2014.29.3.438 PMC3945142

[107] Wilson JG , Liu KD , Zhuo H , et al. Mesenchymal stem (stromal) cells for treatment of ARDS: a phase 1 clinical trial. Lancet Respir Med. 2015;3:24‐32. 25529339 10.1016/S2213-2600(14)70291-7 PMC4297579

[108] Simonson OE , Mougiakakos D , Heldring N , et al. In vivo effects of mesenchymal stromal cells in two patients with severe acute respiratory distress syndrome. Stem Cells Translational Medicine. 2015;4:1199‐11213. 26285659 10.5966/sctm.2015-0021 PMC4572899

[109] Matthay MA , Calfee CS , Zhuo H , et al. Treatment with allogeneic mesenchymal stromal cells for moderate to severe acute respiratory distress syndrome (START study): a randomised phase 2a safety trial. Lancet Respir Med. 2019;7:154‐162. 30455077 10.1016/S2213-2600(18)30418-1 PMC7597675

[110] Yip HK , Fang WF , Li YC , et al. Human umbilical cord‐derived mesenchymal stem cells for acute respiratory distress syndrome. Crit Care Med. 2020;48:e391‐e399. 32187077 10.1097/CCM.0000000000004285

[111] Han J , Li Y , Yuanyuna L . Strategies to enhance mesenchymal stem cell‐based therapies for acute respiratory distress syndrome. Stem Cells Int. 2019;2019:5432134. 31885615 10.1155/2019/5432134 PMC6893276

[112] Russell CD , Millar JE , Baillie JK . Clinical evidence does not support corticosteroid treatment for 2019‐nCoV lung injury. Lancet. 2020;395:473‐475. 32043983 10.1016/S0140-6736(20)30317-2 PMC7134694

[113] Chinese Clinical Trial Registry . A multicenter, randomized controlled trial for the efficacy and safety of tocilizumab in the treatment of new coronavirus pneumonia (COVID‐19). ChiCTR2000029765. Registered February 13, 2020.

[114] Liang B , Chen J , Li T , et al. Clinical remission of a critically ill COVID‐19 patient treated by human umbilical cord mesenchymal stem cells. E‐print. *ChinaXiv 2020:202002.00084*. February 27, 2020. 10.1097/MD.0000000000021429 PMC7402800 32756149

[115] Leng Z , Zhu R , Hou W , et al. Transplantation of ACE2− mesenchymal stem cells improves the outcome of patients with COVID‐19 pneumonia. Aging Dis. 2020;11:216‐228. 32257537 10.14336/AD.2020.0228 PMC7069465

[116] Zhao RC . Stem cell‐based therapy for coronavirus disease 2019. Stem Cells Dev. 2020;29:679‐681. 32292113 10.1089/scd.2020.0071 PMC7247051

[117] Oztürk S , Elçin AE , Elçin YM . Mesenchymal stem cells for coronavirus (COVID‐19)‐induced pneumonia: revisiting the paracrine hypothesis with new hopes? Aging Dis. 2020;11:477‐479. 32489694 10.14336/AD.2020.0403 PMC7220290

[118] Rothan HA , Byrareddy SN . The epidemiology and pathogenesis of coronavirus disease (COVID‐19) outbreak. J Autoimmun. 2020;109:102433. 32113704 10.1016/j.jaut.2020.102433 PMC7127067

[119] Metcalfe SM . Mesenchymal stem cells and management of COVID‐19 pneumonia. Med Drug Discov. 2020;5:100019. 32296777 10.1016/j.medidd.2020.100019 PMC7147223

[120] American Academy of Stem Cell Physicians . The American Academy of Stem Cell Physicians Recommends A Treatment Protocol for COVID‐19 to the WHO. News release. Miami, FL: American Academy of Stem Cell Physicians; 2020.

[121] Turner L . Preying on public fears and anxieties in a pandemic: businesses selling unproven and unlicensed “stem cell treatments” for COVID‐19. Cell Stem Cell. 2020;26:806‐810. 32464095 10.1016/j.stem.2020.05.003 PMC7203029

